# Evaluating the impact of point-of-care HIV viral load assessment on linkage to care in Baltimore, MD: a randomized controlled trial

**DOI:** 10.1186/s12879-023-08459-7

**Published:** 2023-09-01

**Authors:** Mohammad Harris Bayan, Travis Smalls, Alec Boudreau, Agha W. Mirza, Courtney Pasco, Zoe O Demko, Richard E. Rothman, Yu-Hsiang Hsieh, Susan H. Eshleman, Heba H. Mostafa, Nathalie Gonzalez-Jimenez, Pollyanna R. Chavez, Brian Emerson, Kevin P. Delaney, Debra Daugherty, Robin J. MacGowan, Yukari C. Manabe, Matthew M. Hamill

**Affiliations:** 1 Division of Infectious Diseases, Johns Hopkins University, Baltimore, MD USA; 2 Department of Emergency Medicine, Johns Hopkins University, Baltimore, MD USA; 3 Department of Pathology, Johns Hopkins University, Baltimore, MD USA; 4 Centers for Disease Control and Prevention, Atlanta, GA USA; 5 DLH Corporation, Atlanta, GA USA

**Keywords:** HIV, Viral load testing, Diagnostics, Point-of-care, Antiretroviral therapy, Pre-exposure prophylaxis, Linkage-to-care, Randomized clinical trial, Acute HIV infection

## Abstract

**Background:**

Integration of a sensitive point-of-care (POC) HIV viral load (VL) test into screening algorithms may help detect acute HIV infection earlier, identify people with HIV (PWH) who are not virally suppressed, and facilitate earlier referral to antiretroviral therapy (ART), or evaluation for pre-exposure prophylaxis (PrEP). This report describes a randomized clinical trial sponsored by the Centers for Disease Control and Prevention (CDC): “Ending the HIV Epidemic Through Point-of-Care Technologies” (EHPOC). The study’s primary aim is to evaluate the use of a POC HIV VL test as part of a testing approach and assess the impact on time to linkage to ART or PrEP. The study will recruit people in Baltimore, Maryland, including patients attending a hospital emergency department, patients attending an infectious disease clinic, and people recruited via community outreach. The secondary aim is to evaluate the performance characteristics of two rapid HIV antibody tests approved by the United States Food and Drug Administration (FDA).

**Methods:**

The study will recruit people 18 years or older who have risk factors for HIV acquisition and are not on PrEP, or PWH who are not taking ART. Participants will be randomly assigned to either the control arm or the intervention arm. Participants randomized to the control arm will only receive the standard-of-care (SOC) HIV screening tests. Intervention arm participants will receive a POC HIV VL test in addition to the SOC HIV diagnostic screening tests. Follow up will consist of an interim phone survey conducted at week-4 and an in-person week-12 visit. Demographic and behavioral information, and oral fluid and blood specimens will be collected at enrollment and at week-12. Survey data will be captured in a Research Electronic Data Capture (REDCap) database. Participants in both arms will be referred for either ART or PrEP based on their HIV test results.

**Discussion:**

The EHPOC trial will explore a novel HIV diagnostic technology that can be performed at the POC and provide viral assessment. The study may help inform HIV testing algorithms and contribute to the evidence to support same day ART and PrEP recommendations.

**Trial registration:**

NIH ClinicalTrials.gov NCT04793750. Date: 11 March 2021.

**Supplementary Information:**

The online version contains supplementary material available at 10.1186/s12879-023-08459-7.

## Background

Diagnosis of acute human immunodeficiency virus (HIV) infection followed by early antiretroviral therapy (ART) initiation serves both individual and public health, as it has health benefits for the person receiving treatment [1] and viral suppression interrupts onward transmission [2]. During the acute infection period before antibody responses can be detected, HIV antigen or antibody (Ag/Ab) tests may be non-reactive, and therefore, interpreted as negative. The use of tests with improved sensitivity for detecting acute HIV infection through the assessment of HIV viral load (VL) while the patient is in the clinic may facilitate earlier referral for initiation of ART [1, 3]. The use of sensitive HIV VL tests can also facilitate same-day pre-exposure prophylaxis (PrEP) linkage by providing a definitive HIV test result and alleviating the concern of prescribing PrEP for individuals with undiagnosed acute HIV infection [4].

Point-of-care (POC) testing which is defined as near-patient testing performed at home, clinic, doctor’s office, or a hospital has a fast turnaround time [5]; this may range from several minutes to more than an hour. Current HIV testing algorithms in the United States (U.S.) rely on laboratory-based Ag/Ab tests as the first step to detect HIV infection [6]. These tests can detect some early HIV infections but are usually only reactive after the HIV VL has peaked when Ag/Ab are present. HIV VL tests are more sensitive for detection of acute HIV infections and have window periods that are approximately 10-days shorter than most HIV Ag/Ab tests [7]. Routine use of a POC HIV VL test for HIV screening, used sequentially along with Ag/Ab tests, has the potential to improve detection of acute HIV infection and increase early HIV diagnoses. The use of POC HIV VL may also support same-day PrEP linkage by providing fast HIV results and reducing linkage barriers by eliminating the need for multiple visits to a healthcare facility [4].

POC HIV VL tests can also play an important role in the clinical management of HIV. Using these tests has the potential to improve VL monitoring, increase treatment efficacy, and reduce emergence of drug resistance. The adoption of POC HIV VL tests may improve individual health and also reduce transmission to others by facilitating viral suppression in people with HIV (PWH) [8, 9].

A 2019 review of 32 publications examined the performance and clinical utility of POC HIV VL tests [10]. The authors concluded that performance of some POC HIV VL tests was comparable to laboratory-based HIV VL tests, and that use of POC HIV VL tests had the potential to improve patient outcomes. However, only two of the studies evaluated included comparator testing and use of results from POC HIV VL tests to guide clinical management. One of the studies compared the performance of Xpert HIV-1 Quantitative (Quant) VL test to centralized VL testing in Zimbabwe from November 2015 to August 2016. The median ‘days to result delivery to clinician’ using the Xpert HIV-1 Quant VL was 1 day [interquartile range (IQR): 0–2] compared to 27 days (IQR: 23–45) using the conventional and centralized VL testing, with a result concordance rate of 96.8 – 98.5% [11].

Another study, conducted between October 2014 to April 2016 in South Africa, compared the performance of POC Cepheid Xpert HIV-1 Qualitative test to the standard-of-care (SOC) Roche COBAS TaqMan HIV-1 Qualitative test for neonatal HIV diagnosis. The study demonstrated 100% sensitivity [95% Confidence Interval (CI): 88.4–100) and 99.9% specificity (95% CI: 99.7–100). The median time to result return for the POC test was 1 day (IQR: 0–1) as compared to 10 days (IQR: 9–13) for the comparator test (P < 0.0001) [12].

Studies using the Cepheid GeneXpert HIV-1 Qualitative POC HIV VL test to assist with early infant HIV diagnosis in Kenya demonstrated a sensitivity of 94.1% and specificity of 99.8% compared to the SOC Roche CAP/CTM HIV-1 qualitative polymerase chain reaction assay [13].

To assess new HIV testing technologies, it is necessary to evaluate their performance during the acute and early stages of infection and compare their performance with rapid HIV Ag/Ab tests and the gold-standard reference laboratory algorithm. Results of these tests can also help in timely identification of persons with undiagnosed infection, PWH who are not virally suppressed or may need reengagement in care, and those without HIV infection who may benefit from PrEP. This study can also measure the impact of POC HIV VL test results on key clinical decisions including immediate treatment decisions and linking patients to appropriate treatment and/or prevention services.

### Objectives

The primary objective of this trial is to evaluate whether the use of a POC HIV VL test, in addition to SOC HIV testing, for HIV screening increases the proportion of persons linked to ART or PrEP. Secondary objectives are: [1] to compare the performance of rapid HIV antibody tests using fresh whole blood and oral fluid specimens with results obtained using a laboratory-based HIV testing algorithm, [2] to evaluate the sensitivity of HIV antibody tests by testing longitudinal samples, and [3] to compare characteristics of persons with acute HIV infections to those with established infections.

## Methods/design

Ending the HIV Epidemic Through Point-of-Care Technologies (EHPOC) is an unblinded randomized clinical trial that will compare a standard-of-care (SOC) HIV testing algorithm to the SOC algorithm with the addition of POC HIV screening with the Xpert® HIV-1 VL test (Cepheid, Sunnyvale, CA).

### Study sites

Sites planned as part of the study are the Johns Hopkins Hospital Emergency Department (JHHED), the Johns Hopkins Bayview Medical Center, the John G. Bartlett Specialty Practice (JGBSP) Infectious Diseases Clinic, and the Baltimore City Health Department (BCHD) Sexual Health Clinics, all located in a high HIV burden area [14] and an Ending the HIV Epidemic (EHE) jurisdiction in the U.S [15]. The clinical sites were selected to provide optimal settings for patient interaction and access to patients at increased risk of HIV infection or PWH. Each clinical site serves a predominately socioeconomically disadvantaged population; 15% of the adult patient population in the JHHED comprises persons who previously or currently inject drugs, with a high prevalence of sexually transmitted infections (STIs) [16–18].

The study will recruit participants from the community using social media outreach by using social media adverts (Facebook and Instagram) and more traditional methods such as flyers. Community outreach using social media will be critical for access to disproportionately affected populations who are unwilling or unable to engage in traditional healthcare services. Potential participants from community outreach who make contact with research coordinators will be invited to visit the Clinical Research Unit (CRU) of the Institute for Clinical and Translational Research (ICTR) located at the Johns Hopkins Hospital (JHH) where eligibility will be determined, and study visits will be completed. In addition, participants recruited from clinical sites will complete follow-up visits at the CRU.

### Study population

The study will recruit participants over a 36-month period. Each participant will be followed for 12 weeks after enrollment. The study inclusion and exclusion criteria are defined in Table 1 below.

**Table 1:** Eligibility criteria for enrollment into the EHPOC study, Baltimore, MD, 2021–2024

**Inclusion criteria**: - Aged 18 years or older - Persons at high risk for HIV and STIs (e.g., patients who receive STI, HIV and/or hepatitis C virus testing, or treatment for STIs) - People with HIV who do not have a documented undetectable HIV viral load in the past 6 months and are not taking ART as prescribed - Willing to undergo phlebotomy and collection of oral fluid samples - Willing to complete a behavioral and demographic questionnaire - Willing to have laboratory results shared with the clinician(s) and Disease Intervention Specialists (DIS) associated with their care - Willing to attend follow-up visits - Willing to have the research team look at the medical record to record examination findings, diagnoses, and test results when required - Willing to have samples transferred to the Centers for Disease Control and Prevention (CDC) for future analysis and storage	
**Exclusion criteria**: - Aged less than 18 years - People with HIV with documented undetectable HIV viral load in prior 6 months and adherent to ART - Already adherent to PrEP and under clinical follow up - Unwilling to undergo study procedures - Any other reason deemed pertinent by the study team	

ART, antiretroviral therapy; CDC, Centers for Disease Control and Prevention; DIS; disease intervention specialist; HIV, human immunodeficiency virus; PrEP, pre-exposure prophylaxis; STI, sexually transmitted infections

### Recruitment, consent, and randomization

After identification by study coordinators, a clinician will ask potential participants if they are interested or willing to participate in the study. Those who are interested in the study will be introduced to a research coordinator to discuss the study and assess eligibility. Those who do not agree to participate will continue with their routine clinical care. Potential participants who become aware of the study through social media, adverts or peers, will call or email the research coordinators at the phone number or email address provided in the advertisements or posts. If the person is eligible, they will be invited to the CRU.

Informed consent and all other study procedures will be obtained and performed in accordance with the relevant guidelines and regulations of the Johns Hopkins University (JHU) and the Declaration of Helsinki [19]. The participant will be given a copy of the consent document, a copy will be placed in the participant’s medical record, and the original kept in a single, locked cabinet accessible only to the research coordinators of the team.

After obtaining written informed consent, the participant will be randomly assigned, by the study manager or principal investigator, to either the control or the intervention arm and notified of their assignment by the research coordinator. Randomization will be determined using the online software, Sealed Envelope Ltd©, and will be overseen by the study manager.

### Participant removal criteria

Participants may withdraw voluntarily from study participation at any time and those who do not complete all study procedures at enrollment (e.g., do not have blood drawn during visit) will be classified as early withdrawals. Participants who withdraw will continue their care with the appropriate clinical service.

### Enrollment visit

**Fig. 1:**
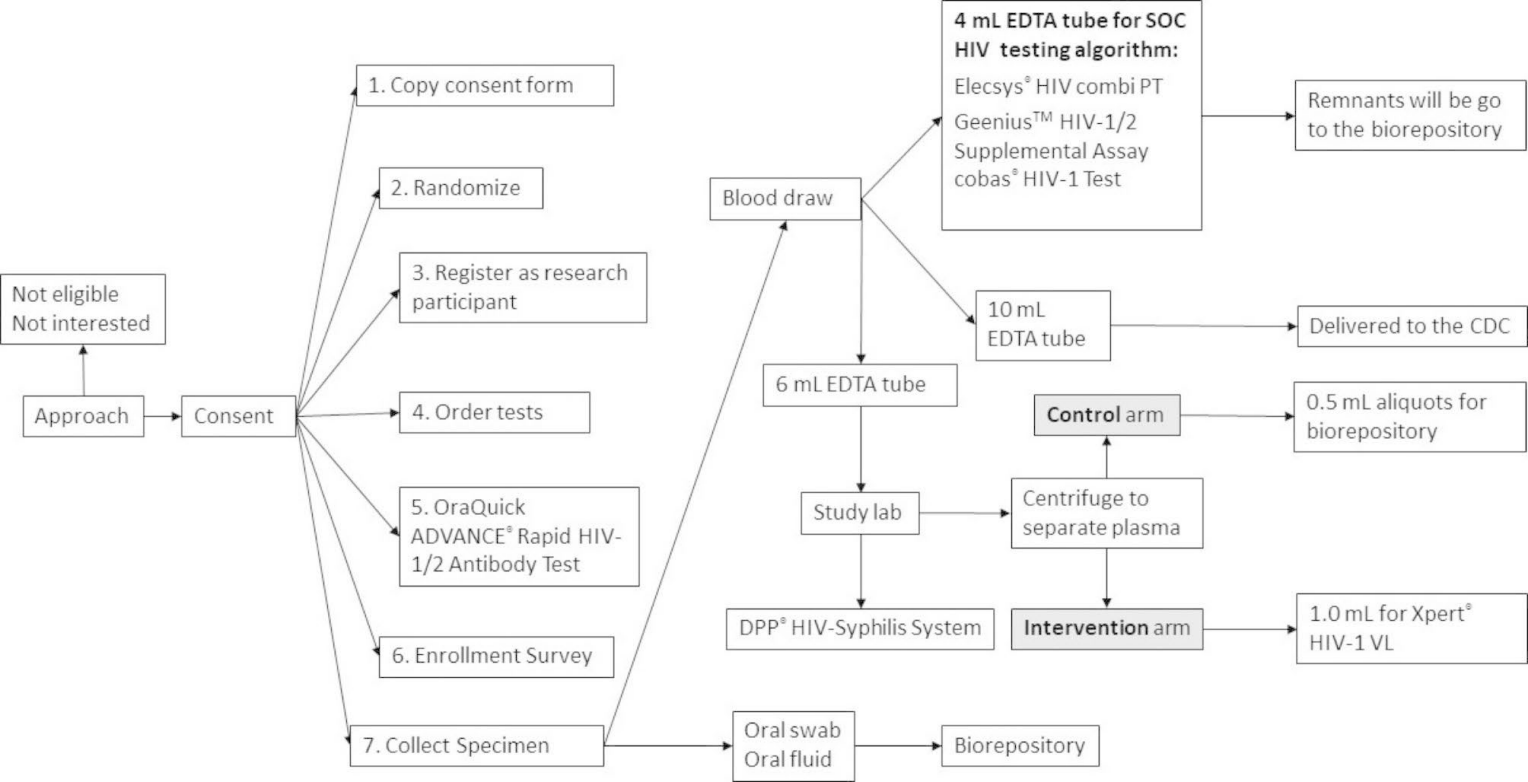
Sequence for enrollment visit procedures in the EHPOC study, Baltimore, MD, 2023–2024 Abbreviations: CDC, Centers for Disease Control and Prevention; EDTA, Ethylenediamine Tetra acetic Acid; HIV, human immunodeficiency virus; mL, milliliter; SOC, standard-of-care; VL, viral load

At the enrollment visit, study participants in both arms will provide blood and oral fluid specimens for HIV testing. The sequence for enrollment visit procedures is presented in Fig. 1. Participants will receive the tests as displayed in Table 2 on the samples they provide. Regardless of study arm, all participants will receive the following tests:

1. OraQuick ADVANCE^®^ HIV-1/2 Rapid Ab test (OraSure Technologies, Bethlehem, PA).
2. DPP^®^ HIV-Syphilis System (Chembio Diagnostic Systems, Inc., Medford, NY).
3. Elecsys^®^ HIV combi PT (Roche Diagnostics, Indianapolis, IN).
4. Geenius^™^ HIV-1/2 Supplemental Assay (Bio-Rad Laboratories, Inc., Redman, WA) if Elecsys^®^ HIV combi PT is reactive.

**Table 2:** HIV Laboratory and POC tests performed in the EHPOC study, Baltimore, MD, 2023–2024

Test name	Test description	Indication/use	Participants tested	FDA Approved	Specimen	Manufacturer
Xpert® HIV-1 VL	Rapid quantitation of HIV-1 RNA	This test will be in addition to the SOC algorithm as an HIV screening test - 90 min TAT	Participants in the intervention arm	No	Plasma	Cepheid, Sunnyvale, CA
Elecsys® HIV combi PT	Simultaneous qualitative detection and differentiation of HIV p24 Ag and Ab to HIV-1 and HIV-2	HIV screening in the SOC algorithm - Variable TAT (mostly within 24 h)	All participants	Yes	Plasma	Roche Diagnostics, Indianapolis, IN
Geenius™ HIV-1/2 Supplemental test	Confirm the presence of Ab to HIV-1 and HIV-2	HIV confirmation in the SOC algorithm - Variable TAT (mostly within 24 h)	Participants with a reactive Elecsys HIV combi PT Ag/Ab test	Yes	Plasma	Bio-Rad Laboratories, Inc., Redman, WA
cobas® HIV-1 Test	Quantitation of HIV-1 RNA	HIV confirmation in the SOC algorithm and comparator to the Xpert® HIV-1 VL test - Variable TAT (mostly within 48 h)	Participants with a negative or indeterminate Geenius test and all participants in the intervention arm.	Yes (for monitoring HIV VL)	Plasma	Roche Molecular Systems, Inc, Pleasanton, CA
OraQuick ADVANCE® Rapid HIV-1/2 Antibody Test	Rapid test for detection of Ab to HIV-1 and HIV-2	HIV screening and Comparison of rapid HIV antibody tests - 20 min TAT	All participants	Yes	Oral fluid	OraSure Technologies, Bethlehem, PA
DPP® HIV-Syphilis System	Rapid test for detection of Ab to HIV-1 and HIV-2	HIV screening and Comparison of rapid HIV antibody tests - 20 min TAT	All participants	Yes	Whole blood	Chembio Diagnostic Systems, Inc., Medford, NY

Abbreviations: Ag: antigen; Ab, antibodies; CA, California; HIV, human immunodeficiency virus; IN, Indiana; NY, New York; FDA: Food and Drug Administration; PA, Pennsylvania; RNA ribonucleic acid; SOC, standard-of-care; TAT, turnaround time; VL, viral load; WA, Washington.

Oral fluid samples and plasma aliquots will be processed and frozen for storage in a biorepository for future HIV test evaluation purposes (Fig. 1).

The SOC HIV testing algorithm will be performed at the Immunology Laboratory at JHH. The current SOC HIV testing algorithm consists of the following tests: a laboratory-based, instrumented, Elecsys® HIV combi PT (Roche Diagnostics, Indianapolis, IN), the Geenius™ HIV-1/2 Supplemental Assay (Bio-Rad Laboratories, Inc., Redman, WA), with reflex, when appropriate, to the cobas® HIV-1 test (Roche Molecular Systems, Inc, Pleasanton, CA) (See Table 2).

The study will collect participants’ demographic data, as well as sexual, and drug use behaviors. Additionally, their medical records will be examined to document physical exam findings.

In addition to the procedures described previously, participants assigned to the intervention arm will be tested using a research use only POC HIV VL test (Xpert® HIV-1 VL) during the enrollment visit. This will be supplemented by the cobas® HIV-1 test, which will be available for clinical use (Table 3).

**Table 3:** Schedule of events in the EHPOC study, Baltimore, MD, 2023–2024

Study Visit	Enrollment		Week-4		^1^Additional Follow up		Week-12	
Study Arm	C	I	C	I	C	I	C	I
Informed Consent	X	X						
Randomization	X	X						
Questionnaires/Surveys	X	X	X	X	X	X	X	X
Oral swab and oral fluid collection	X	X			X	X	X	X
Blood draw for biorepository	X	X			X	X	X	X
OraQuick ADVANCE® Rapid HIV-1/2 Antibody Test	X	X			X	X		
DPP® HIV-Syphilis Test	X	X			X	X		
Elecsys® HIV Combi PT Geenius™ HIV-1/2 Supplemental Assay (see Table 2)	X	X			X	X		
Xpert® HIV-1 Viral Load		X				X		
cobas® HIV-1 Test		X			X	X	X	X
Review of Medical Records	X	X	X	X	X	X	X	X

Abbreviations: Ab, antibody; Ag, antigen; C, Control arm; HIV, human immunodeficiency virus; I, Intervention arm; SOC, standard of care; POC, point-of-care

1) Participants whose POC VL or rapid test result is discordant with laboratory-based test results will be offered additional study follow-up visits. The additional follow-up schedule will include return visits at Days 3, 7, 10, 14, 21, 28, 42, 56, and 70 after the enrollment visit. The participants with discordant results will remain in the study until one of the following events occurs:

a) Participant tests reactive/positive for HIV for all tests being evaluated, or

b) Participant has two consecutive study visits with non-reactive/negative results for all tests (indicating that they had a prior false-reactive/positive result)

### Week-4 and week-12 visits

All participants will be followed-up post enrollment (Table 3). The Week-4 visit will take place 4 weeks (-7 to + 14 days) post-enrollment, and will be completed over the phone. The Week-12 (-14 to + 21 days) post-enrollment visit will be completed in-person. At these visits, participants will be asked about their sexual behavior and drug use since the enrollment visit. Additionally, they will be asked about HIV-related symptoms, about any healthcare received since their enrollment visit, and for information about linkage-to-care (LTC) activities they have received. The research coordinators will review participant’s medical records to document new physical exam findings, results of interval laboratory results, and changes in medication.

### Discordant result visits

Participants who have discordant results between (a) the POC HIV VL test or (b) the rapid HIV tests compared with the laboratory-based HIV tests will be offered additional study follow-up visits to evaluate the sensitivity of different HIV tests for detection of early HIV infection.

The participants with discordant results will remain in the study until one of the following events occurs:

1. Participant tests reactive/positive for HIV for all tests being evaluated,
2. Participant has two consecutive study visits with non-reactive/negative results for all tests (indicating that they had a prior false-reactive/positive result), or
3. Participant completes follow-up of 70 days after the enrollment visit

The follow-up schedule for those with discordant results will include return visits at Days 3, 7, 10, 14, 21, 28, 42, 56, and 70 post-enrollment visits. At each follow-up visit, participants will provide blood and oral fluid specimens for storage and testing along with the rapid HIV tests under evaluation (e.g., OraQuick ADVANCE® Rapid HIV-1/2 Antibody test, DPP® HIV-Syphilis System); other laboratory HIV diagnostics where applicable (Elecsys® HIV combi PT, Geenius™ HIV-1/2 Supplemental Assay), and the cobas® HIV-1 test (Table 3). The same questionnaires and medical record review as for the Week-4 and Week-12 visits will be undertaken at these visits.

### Follow up after the enrollment clinic visit and test results

**Fig. 2:**
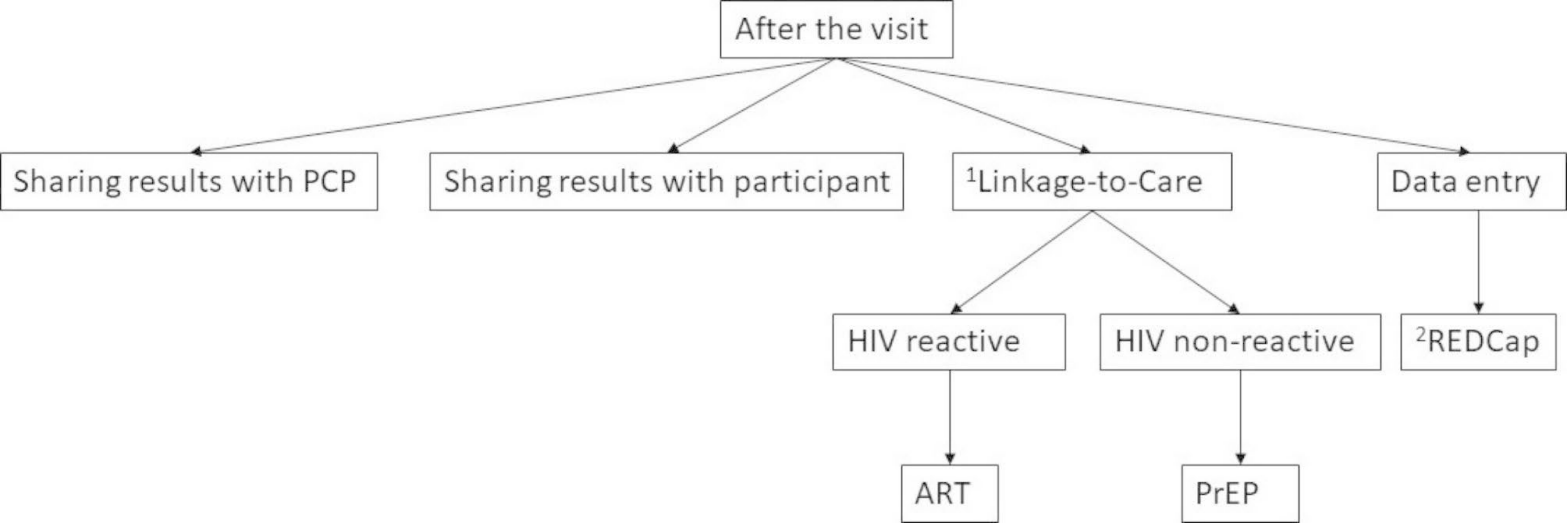
Follow up activities after a participant’s enrollment visit in the EHPOC study, Baltimore, MD Abbreviations: ART, antiretroviral therapy; PCP, primary care provider; PrEP, preexposure prophylaxis; REDCap, Research Electronic Data Capture 1) Participants will be referred to the following sites for Linkage-to-Care: Baltimore City Health Department, the John G. Bartlett Specialty Practice, Johns Hopkins Bayview Medical Center. These sites are located in Baltimore, Maryland and will be accessible to study participants 2) REDCap is Research Electronic Data Capture software used to store study data

Within 24 h of results being available, research coordinators will communicate HIV test results to the participants and share the results with the participant’s primary care provider. Key clinical decisions that are impacted by having the results of the POC HIV VL test will be recorded based on participant interviews and medical chart review. If the participant is still in the clinic when the POC HIV VL test results are obtained, study coordinators will initiate LTC activities. Participants with confirmed HIV infection will be referred by the research coordinators to their preferred clinician. Those with newly diagnosed HIV infection will be referred to the local Disease Intervention Specialist team for additional LTC and partner services. Participants with non-reactive (negative) test results will be referred to their preferred clinician for consideration of HIV PrEP and prevention counseling (Fig. 2). Participants who are not present when their results are available will be contacted within 24 h of results being available to discuss the results. Three separate attempts will be made to contact participants to communicate the results and facilitate LTC.

### Specimen and data collection

Two biospecimens will be collected: oral (swab and fluid), and blood via venipuncture. The research coordinators will be trained in phlebotomy by the ICTR and follow Good Clinical Practice (GCP). In addition, trained phlebotomists will be available for venipuncture for participants attending the CRU. Samples will be transported and delivered to JHH laboratories for testing, processing, and storage using standard operating procedures.

Survey questionnaires will be conducted using an iPad and answers will be stored in Research Electronic Data Capture (REDCap). The data will be accessed by the research coordinators and team members of the study only using JHU workstations or other computers using the JHU SAFE Desktop feature. The SAFE Desktop is a secure cloud-based platform, which is fully Health Insurance Portability and Accountability Act (HIPAA) compliant and managed by JHU. Examination of participant medical charts will be conducted using JHU workstations or using the SAFE Desktop feature. A group of designated JHU faculty will have responsibility for monitoring, oversight of adverse events and other protocol events.

### Outcome measures

The primary outcome for this trial is the proportion of participants that are linked either to ART or PrEP. Linkage is defined as having at least one interaction (a telehealth or in-person conversation about HIV PrEP or ART) with a healthcare provider during the 12-week follow up period after enrollment. After the Week-12 visit, participants will continue to receive SOC services as appropriate. The secondary outcomes include time to linkage to care for HIV ART or PrEP; HIV case identification; proportion of PWH started on ART; proportion of PWH who are on ART and are virally suppressed at 12 weeks, and self-reported behavioral change since the time of diagnosis.

### Power calculation and analysis plan

Using data from prior studies, we observed an LTC rate of 22% for the JHHED patients who are living with HIV but not in care or who are HIV-negative and PrEP-eligible, and an LTC rate of 50% for PrEP-eligible patients attending STI services or recruited via social media, resulting in an overall aggregate LTC rate of 32.5% (with 63% patient enrollment in the JHHED and 37% in the STI clinics/participants recruited via social media). To detect an increase of LTC rate from 32.5 to 52.5% within the intervention arm, with 80% power and a two-sided significance level of 0.05, a total of 95 participants are required in each arm at the end of the study if the same proportion of participants are enrolled from JHHED. Factoring in a 15% lost-to-follow-up, 112 participants will be enrolled into each arm.

We will use a bivariate analysis to explore the potential association between the independent variable (POC HIV VL test) and other covariates with the outcome variable of interest. A multivariate regression is planned to determine the association between having a POC HIV VL test and linkage to PrEP or ART, with adjustment for covariates and confounders. A survival analysis will be performed (Cox proportional hazard model) to model the time to linkage to ART or PrEP by study arm, with adjustment of covariates and confounders.

The performance of the OraQuick ADVANCE® Rapid HIV-1/2 Antibody Test and the DPP® HIV-Syphilis System will be evaluated by calculating the positive percent agreement, negative percent agreement, positive predictive value (PPV), and negative predictive value (NPV) compared to the standard laboratory HIV testing algorithm.

### Reporting unanticipated problems or study deviations

Any protocol deviation or adverse event that meets reporting requirements of the Institutional Review Board (IRB) will be reported to the Johns Hopkins IRB, and a de-identified report will be submitted to the CDC. All deviations from the protocol will be addressed in the study participant source documents. The documentation will include the reason(s) for the deviation and all attempts made to prevent or correct the deviation. The study team will complete a Protocol Deviation Form documenting each protocol deviation. A complete copy of the Protocol Deviation Form will be maintained in the regulatory file as well as in the participant’s source documents.

### Benefits of participation

Participants may benefit from earlier diagnosis of HIV, which should facilitate earlier treatment and avoidance of the sequalae of untreated infection. Participants will have the opportunity to learn more about their sexual health and will be referred for rapid initiation of HIV treatment and prevention services, as well as risk reduction counselling with a clinician. There will be no cost to the participants for study-related activities or tests conducted, participants will receive a reimbursement of $40 for each in-person study visit completed.

Society may benefit through (a) the reduction of transmission of HIV in the community; (b) findings may inform CDC HIV testing guidelines for clinical settings.

## Discussion

This clinical trial may demonstrate that HIV testing algorithms using a POC HIV VL screening test has the potential to facilitate same-day initiation of ART or PrEP and improve the diagnosis of acute HIV infection, which may be missed using existing HIV testing algorithms. Detection of acute HIV infection is especially important, since persons with acute HIV infection often have high viral loads which are associated with high transmission risk. Accurately diagnosing HIV infections with POC HIV VL screening tests could facilitate counseling and LTC while the patient is still in the clinical encounter. Having accurate real-time information on the HIV VL in PWH may also impact key treatment decisions, particularly in acute and episodic care settings. Further, having real-time results has the potential to reduce delays in ART initiation and reduce loss to follow up. Finally, assessment of HIV status among those at risk for HIV infection using a POC HIV VL test could also expedite referral for care and initiation of PrEP.

Same-day ART recommendation has the potential to improve patient health outcomes. In a prior Haitian study, 347 participants were enrolled in the same-day ART group and 356 participants were enrolled in standard ART group. The results of the study showed that the probability of remaining in care 12 months post enrollment for participants in the same-day ART group was higher (risk ratio 1.21) than for those in the standard ART group (p = 0.015) [20]. The EHPOC trial will evaluate the use of a POC HIV VL test for facilitating rapid initiation of referral to ART and add to the literature through its comparison to the gold standard laboratory HIV VL test and SOC HIV testing algorithm.

In high-income settings like the US, more evidence supporting same day ART initiation is needed. The Panel on Antiretroviral Guidelines for Adults and Adolescents [21] recommends initiating ART at the time of diagnosis (when possible) or soon afterwards to increase the uptake of ART, decrease the time required to achieve LTC and virologic suppression, and improve the rate of virologic suppression among individuals who have recently received HIV diagnoses [21]. The recommendation is rated strong, and based on data from well-designed nonrandomized trials or observational cohort studies with long-term clinical outcomes. The results from EHPOC, a randomized clinical trial, will contribute to the evidence on same day ART recommendations.

Same-day PrEP initiation may encourage engagement in care, reduce loss to follow-up, and reduce linkage barriers by eliminating the need for multiple visits to a healthcare facility. A barrier to same-day PrEP initiation is the possibility of prescribing PrEP for individuals with undiagnosed, acute HIV infection based on the results of rapid tests with imperfect sensitivity. This may lead to antiretroviral resistance. To minimize this risk, and to provide reassurance to patients and providers, sensitive HIV VL tests can be used to provide a definitive HIV test result before prescribing PrEP [4]. The EHPOC trial will evaluate the use of a POC HIV VL test and contribute to the evidence on same-day PrEP prescription.

EHPOC study sites, located in a high HIV burden area and an EHE jurisdiction, serve historically socio-economically disadvantaged populations and are ideal locations to serve the Baltimore City community members, including individuals who have no insurance or are underinsured, and marginalized populations that often do not have equity of access to research.

### Trial Status

Recruitment of participants is planned to begin in Spring, 2023.

### Electronic supplementary material

Below is the link to the electronic supplementary material.

Supplementary Material 1

## Data Availability

Not applicable.

## Abbreviations

Ab: Antibody
Ag: Antigen
ART: Anti-Retroviral Therapy
BCHD: The Baltimore City Health Department
C: Control arm
CA: California
CI: Confidence Interval
CDC: Centers for Disease Control and Prevention
CRU: Clinical Research Unit
DIS: Disease Intervention Specialist
EDTA: Ethylenediamine Tetraacetic Acid
EHE: Ending the HIV Epidemic
EHPOC: Ending the HIV Epidemic Through Point-of-Care Technologies
FDA: Food and Drug Administration
GCP: Good Clinical Practice
HIPAA: Health Insurance Portability and Accountability Act
HIV: Human Immunodeficiency Virus
I: Intervention arm
IN: Indiana
ICTR: Institute for Clinical and Translational Research
IQR: Interquartile Range
IRB: Institutional Review Board
JGBSP: The John G. Bartlett Specialty Practice
JHH: Johns Hopkins Hospital
JHHED: The Johns Hopkins Hospital Emergency Department
JHU: Johns Hopkins University
LTC: linkage to care
MD: Maryland
NY: New York
PA: Pennsylvania
PCP: Primary care provider
POC: Point of Care
PrEP: Pre-Exposure Prophylaxis
PWH: People with HIV
Quant: Quantitative
REDCap: Research Electronic Data Capture
RNA: Ribonucleic Acid
STI: Sexually Transmitted Infections
SOC: Standard of Care
TAT: Turnaround time
US: United States
VL: Viral Load
WA: Washington
